# Effects of dietary berberine hydrochloride on growth, immunity, meat quality, and fecal microbiota in broiler chickens

**DOI:** 10.3389/fvets.2026.1766777

**Published:** 2026-02-18

**Authors:** Juan Chen, Changxu Lv, Mingyang Tan, Desheng Li, Zhaoquan Fu, Yaping Wang, Qiangqiang Zou

**Affiliations:** 1 Health & Medical Sciences College, Yantai Nanshan University, Yantai, China; 2 School of Medical and Health Care, Shandong Cultural Industry Vocational College, Qingdao, China; 3 College of Animal Husbandry and Veterinary Medicine, Jinzhou Medical University, Jinzhou, China

**Keywords:** AA+ broilers, berberine hydrochloride, fecal microbiota, growth performance, meat quality

## Abstract

This study aimed to explore the impact of dietary berberine hydrochloride (BBH) on 600 one-day-old AA+ broilers. The broilers were randomly allocated into five groups: a control group (TR) and four experimental groups supplemented with 25, 50, 100, or 200 mg/kg of BBH (designated as Ber25, Ber50, Ber100, and Ber200, respectively). The 42-day experiment consisted of six replicates per group. The results indicated that from 1 to 21 days of age, BBH had no significant influence on growth parameters such as body weight gain (BWG), feed intake (FI), and feed conversion ratio (FCR). However, during 22–42 days and 1–42 days, 50-mg/kg BBH (Ber50) significantly increased BWG and FI, showing a quadratic effect. BBH linearly enhanced the spleen and bursa indices, with the spleen index in the Ber200 group higher than that in the Ber25 group. In 42-day-old broilers, the Ber200 group had the highest levels of antibodies against Newcastle disease and avian influenza H9, presenting both linear and quadratic effects. The Ber100 treatment maximized the pectoralis CIE L* value, and the Ber200 treatment increased the shear force. BBH decreased fecal *Salmonella* counts, demonstrating linear and quadratic effects, and it had a linear impact on *Lactobacillus* counts, although no inter-group differences were observed. In conclusion, 50 mg/kg of BBH improved broiler growth performance, while higher doses such as 200 mg/kg enhanced immunity, reduced *Salmonella* levels, but also increased meat shear force.

## Introduction

In modern broiler production, antibiotics have been extensively utilized for their growth-promoting and intestinal disease-preventing properties to meet the escalating demand for animal protein (1). However, their long-term and irrational application has led to various issues, including antibiotic residues, bacterial resistance, and environmental pollution, thereby posing a substantial threat to public health (2, 3). Consequently, the development of safe and efficacious alternatives to antibiotics has become a critical imperative. Natural plant extracts, particularly traditional Chinese medicinal herbs, have emerged as promising substitute candidates owing to their natural origin, low residue levels, and minimal risk of inducing bacterial resistance (4, 5).

Berberine hydrochloride (BBH) is a natural isoquinoline alkaloid extracted from medicinal herbs such as *Coptis chinensis* and *Phellodendron amurense* (6). Studies have demonstrated that BBH possesses a diverse array of biological activities, including antibacterial effects against intestinal pathogens such as *Salmonella* and *Escherichia coli* (7, 8), improvement of intestinal health via the regulation of intestinal barrier function and gut microbiota structure (9), and participation in host immune modulation to promote immune organ development and antibody production (7, 10). Furthermore, BBH may also influence the utilization efficiency of nutrients by regulating metabolic processes (11). These properties endow BBH with substantial potential value as a green feed additive in livestock and poultry production.

In broiler production, preliminary studies have investigated the effects of BBH on growth performance and health (12–14). However, the majority of existing studies have focused on a single dose or a limited set of indicators, and the evaluation of the multi-dose, systematic effects of BBH in AA+ broilers remains incomplete. In particular, systematic research on the comprehensive impacts of BBH on growth performance, immune response, meat quality, and fecal microbiota is still lacking; its optimal supplemental dose and the potential adverse effects of high doses on meat quality remain to be elucidated. Furthermore, the majority of studies have not performed correlative analyses between immune enhancement and changes in production indicators (e.g., growth and meat quality), which hinders the development of precision application strategies that balance production efficiency and product quality in commercial production.

Against this background, the present study was conducted using 1-day-old AA+ broilers as experimental animals, aiming to systematically investigate the effects of dietary supplementation with different levels of berberine hydrochloride (BBH; 25, 50, 100, and 200 mg/kg) on growth performance, immune organ indices, specific antibody levels, key meat quality indicators, and the abundance of specific bacteria in feces. By clarifying the dose–response relationship of BBH, this study seeks to determine its optimal supplemental level in AA+ broiler production and to provide a scientific basis for BBH as a green feed additive that can synergistically improve growth, immunity, and intestinal health.

## Materials and methods

### Animals and experimental design

In this study, 600 one-day-old AA+ white-feather broilers (a mixture of male and female chickens) were selected as research objects. These broilers were randomly allocated into 5 groups, with each group consisting of 6 replicate pens, and each pen housing 20 broilers. The control group was fed a basal diet (designated as TR). In contrast, for the four experimental groups, namely Ber25, Ber50, Ber100, and Ber200, 25, 50, 100, and 200 mg/kg of BBH (berberine hydrochloride) was added to the basal diet, respectively.

The trial spanned 42 days and was divided into 2 stages: the 1–21-day-old stage and the 22–42-day-old stage. This study aimed to explore the effects of different BBH addition levels on the diet. Berberine hydrochloride (BBH) used in this study has a chemical formula of C₂₀H₁₈ClNO₄ and a molecular weight of 371.81 g/mol. It appeared as a yellow crystalline powder with an active ingredient content of 98.5% and was purchased from Liaoning Kaiwei Biotechnology Co., Ltd. (Liaoning Province, China).

### Animal feeding management

All birds were housed in pens in an experimentally controlled room. The temperature in the room started at 33 °C and decreased by 3 °C every week until it reached 22 °C, with a relative humidity of 65%. During the experiment, the birds had free access to feed and water. The diets were formulated to meet the nutrient requirements recommended by the NRC (15) and were provided in mashed form (Table 1). The broilers were vaccinated against Newcastle disease and avian influenza on the seventh day after hatching. On the 21st day after hatching, the vaccine against Newcastle disease was administered through water.

**Table 1:** Composition and nutrient levels of the experimental basal diet (%, as-fed basis).

Ingredients^1^, %	Days 1–21	Days 22–42
Corn	59.05	57.75
Soybean oil	2.00	6.00
Corn gluten meal (60%, CP)	4.00	4.00
Soybean meal (45%, CP)	30.5	28.50
Limestone	1.40	1.00
Dicalcium phosphate	1.30	1.00
Salt	0.25	0.25
Premix	1.50	1.50
Total	100	100
Analyzed composition, %		
Metabolizable energy (Kcal/kg)	2,900	3,100
Crude protein	23.00	21.00
Calcium	0.90	0.75
Total phosphorus	0.60	0.50

^1^Provided per kg of complete diet: VA 10,000 IU; VD₃ 4,000 IU; VE 40 IU; VK 4 mg; VB₁ 5 mg; VB₂ 8 mg; niacin 65 mg; pantothenic acid 20 mg; VB₆ 5 mg; biotin 0.5 mg; folic acid 2 mg; VB₁₂ 0.02 mg; Cu 9 mg; Fe 85 mg; Zn 78 mg; Mn 0.6 mg; I 0.6 mg.

### Sampling and measurements

#### Growth performance

Before weighing on days 21 and 42, the broilers were fasted for 12 h. Daily feed intake was measured in duplicate and recorded. For each broiler, body weight gain (BWG), feed intake (FI), and feed conversion ratio (FCR) were calculated.

### Organ index

Upon conclusion of the 42-day trial, one broiler was randomly selected from each replicate pen, amounting to a total of six broilers (three male and three female broilers). Following a 12-h fasting period, their live body weights were measured. Subsequently, the selected broilers were humanely euthanized. Euthanasia was carried out by inhaling 100% CO_2_ for 5 min followed by cervical artery bleeding. Blood was then collected from the carotid artery for serum preparation. Immediately after the blood collection, the spleen, thymus, and bursa of Fabricius were carefully dissected. The surface blood of these organs was blotted dry using filter paper, and any surface fat and connective tissues were meticulously removed before weighing. Finally, the organ indices were calculated.

### Blood indicators

Blood samples collected from 42-day-old broilers were left at room temperature for 30 min and centrifuged at 1200 r/min for 15 min to extract the supernatant. The potency of serum antibodies to Newcastle disease and avian influenza H9 is determined by a hemagglutination inhibition test (16). Specifically, the hemagglutination (HA) assay was first performed to determine the hemagglutination titer of the antigen, based on which the viral antigen with four hemagglutination units (4HAU) was prepared. Subsequently, the hemagglutination inhibition (HI) assay was conducted for detection: sample sera were added to U-bottom hemagglutination plates and subjected to 2-fold serial dilution. The prepared 4HAU viral antigen was then added, followed by incubation at 37 °C for 30 min. After this process, 1% chicken red blood cell suspension was added, and incubation was continued for 20–30 min before observing the results. Through this process, specific antibodies against Newcastle disease virus (NDV) and avian influenza virus subtype H9 (H9 AIV) in the samples were determined.

### Meat quality

Upon euthanasia of the broilers at 42 days of age, specimens of their pectoralis major muscles were collected for meat quality assessment. The values of CIE L*, CIE a*, CIE b*, pH, and shear force of the broilers’ pectoralis major muscles were measured in accordance with the methodology proposed by Lee et al. (17).

### Fecal bacterial counts

A 1 g sample of broiler manure from each replicate was collected weekly and transported on ice to the laboratory following the method described by Dang et al. (18). Each replicate of the 1 g fecal sample was diluted and mixed with 9 mL of 1% peptone broth. The viable counts of *E. coli*, *Lactobacillus,* and *Salmonella* in fecal samples were determined on McConkey agar plates, MRS agar plates, and BS agar plates (in 10 g/L of peptide solution) in a biosafety cabinet. The microbial count is ultimately expressed as log_10_ colony-forming units per gram of feces.

### Data analysis

The experimental data were arranged in a completely randomized grouping design. Each replicate pen served as an experimental unit. For the means showing significant differences, multiple comparisons were carried out using the one-way ANOVA LSD method in SPSS 25.0, and visualization was achieved using GraphPad Prism 10. Data are presented as mean ± SD (standard deviation). A *p*-value less than 0.05 was considered to indicate a significant difference. Additionally, analysis was conducted using linear and quadratic terms in contrast.

The results of the correlation analysis were obtained and presented using R (V4.5.1). For the 42-day indicators, Pearson’s correlation analysis was performed using the corrplot, ggplot2, and GGally packages in R (V4.5.1). A significant correlation was determined when *p* < 0.05 and either *R* > 0.6 or *R* < − 0.6.

## Results

### Growth performance

**Figure 1:**
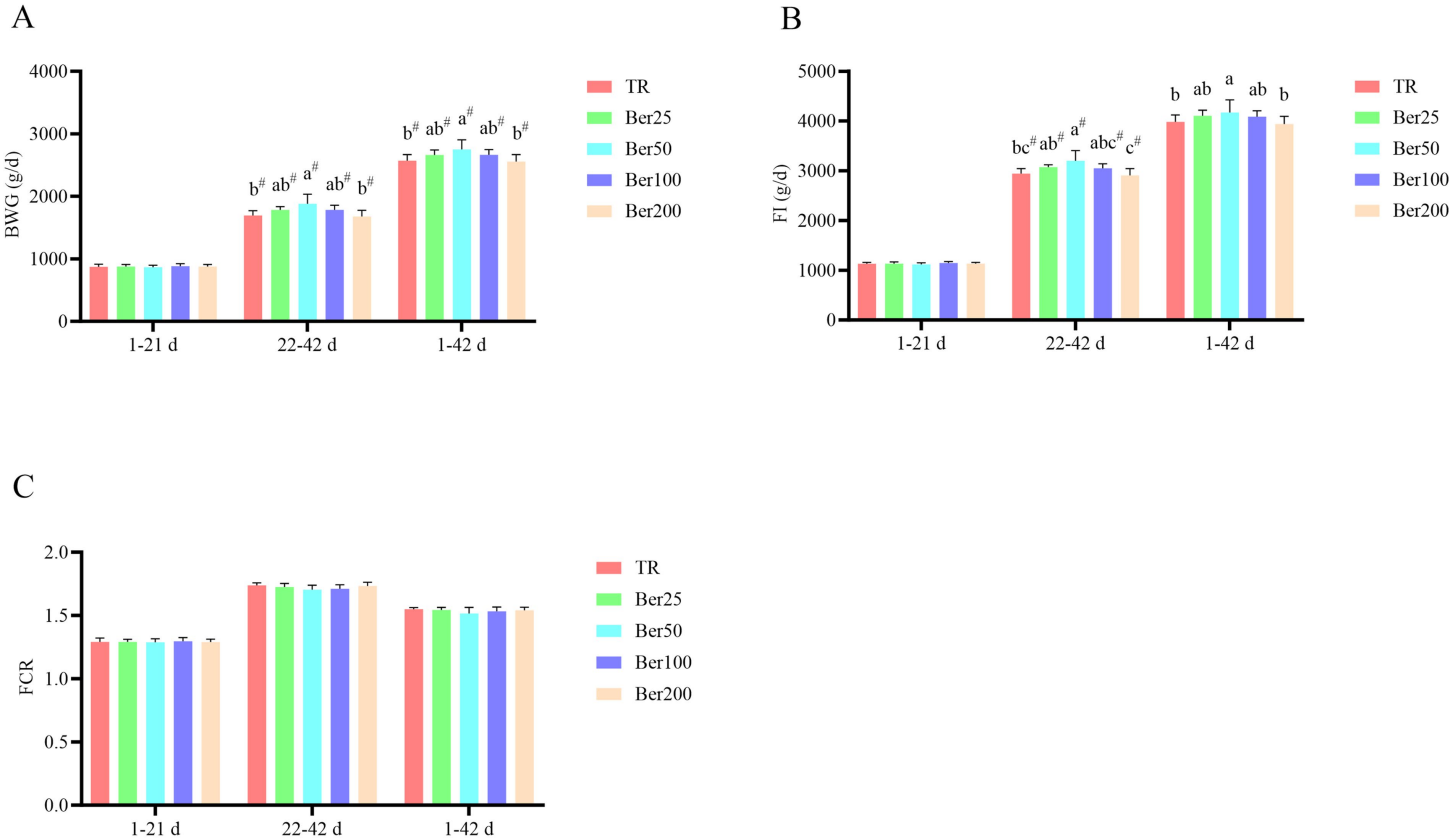
Impact of BBH on the growth performance of AA+ broilers. **(A)** Body weight gain, **(B)** feed intake, and **(C)** feed conversion ratio. ^a–c^Different superscripts in different groups indicate significant differences (*p* < 0.05). # indicates a significant quadratic effect among these groups (*p* < 0.05). No “a–c” or “#” indicates that the difference is not significant (*p* > 0.05). The pen was considered the experimental unit for all analyses (*n* = 6).

As depicted in Figure 1, from 1 to 21 days, supplementation of various dosages of berberine hydrochloride (BBH) in the diet exerted no significant impacts on the body weight gain (BWG), feed intake (FI), and feed conversion ratio (FCR) of broilers (*p* > 0.05). From 22 to 42 days, compared with the control group (TR), inclusion of 50 mg/kg BBH (Ber50) in the diet led to a significant elevation in both BWG and FI of broilers, with a quadratic effect evident (*p* < 0.05). Over the entire 1–42-day period, dietary BBH supplementation manifested a quadratic effect on broiler BWG, with the Ber50 group showing the highest value (*p* < 0.05). Furthermore, the FI of broilers in the Ber200 group was significantly lower than that in the Ber50 group (*p* < 0.05).

### Organ index

**Figure 2:**
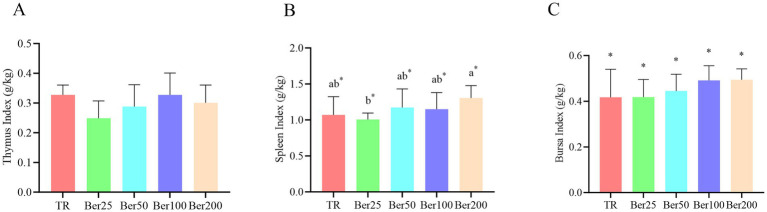
Impact of BBH on the organ index of AA+ broilers. **(A)** Thymus index, **(B)** spleen index, and **(C)** bursa index. ^a,b^Different superscripts in different groups indicate significant differences (*p* < 0.05). “*”indicates a significant linear effect among these groups (*p* < 0.05). No “a,b” or “*” indicates that the difference is not significant (*p* > 0.05). The pen was considered the experimental unit for all analyses (*n* = 6).

As depicted in Figure 2, dietary supplementation of varying dosages of BBH linearly enhanced the spleen index and bursa index in broilers (*p* < 0.05). Nevertheless, the inter-group differences in the bursa index did not attain a significant level (*p* > 0.05). As for the spleen index, a significant disparity was observed between the Ber200 group and the Ber25 group (*p* < 0.05).

### Blood indicators

**Figure 3:**
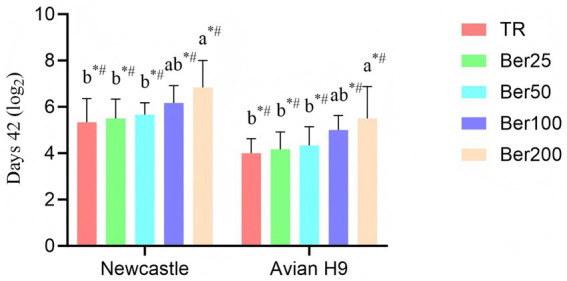
Impact of BBH on the blood indicators of AA+ broilers. Antibody levels in serum at 42 days. ^a,b^Different superscripts in different groups indicate significant differences (*p* < 0.05). “*” indicates a significant linear effect among these groups (*p* < 0.05). “#” indicates a significant quadratic effect among these groups (*p* < 0.05). No “a,b,” “*,” or “#” indicates that the difference is not significant (*p* > 0.05). The pen was considered the experimental unit for all analyses (*n* = 6).

As depicted in Figure 3, dietary supplementation of varying dosages of BBH induced both linear and quadratic effects on Newcastle disease and avian H9 antibody levels in the serum of 42-day-old broilers (*p* < 0.05). Specifically, the Ber200 broilers exhibited the highest antibody levels in their serum.

### Meat quality

**Figure 4:**
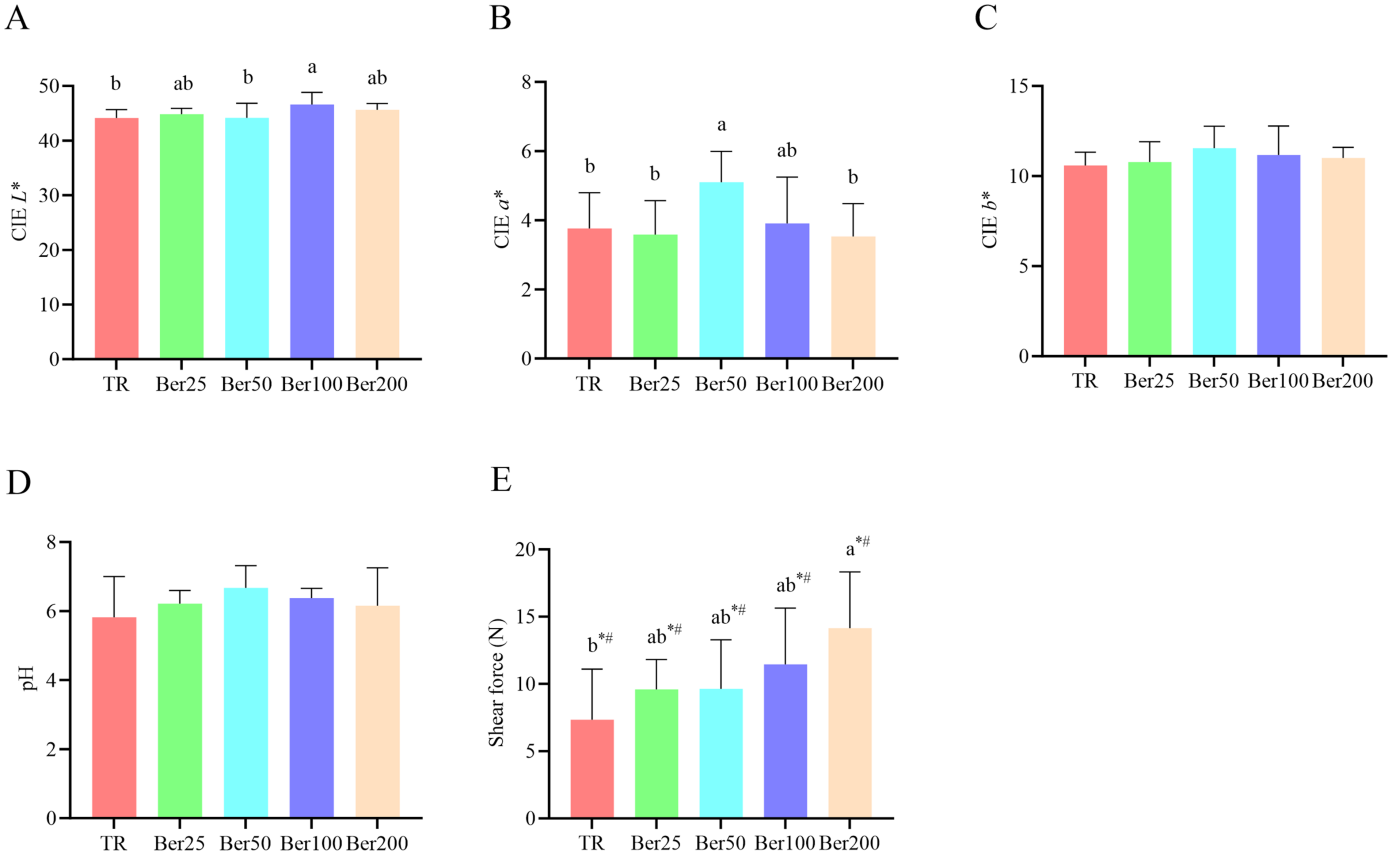
Impact of BBH on the meat quality of AA+ broilers. **(A)** CIE L*, **(B)** CIE a*, **(C)** CIE b*, **(D)** pH, and **(E)** shear force. ^a,b^Different superscripts in different groups indicate significant differences (*p* < 0.05). “*” indicates a significant linear effect among these groups (*p* < 0.05). “#” indicates a significant quadratic effect among these groups (*p* < 0.05). No “a,b,” “*,” or “#” indicates that the difference is not significant (*p* > 0.05). The pen was considered the experimental unit for all analyses (*n* = 6).

As depicted in Figure 4, dietary supplementation with BBH significantly elevated the CIE L* value of broiler breast muscle. Among all groups, the Ber100 group exhibited the maximum CIE L* value (*p* < 0.05). Moreover, BBH supplementation induced both linear and quadratic effects on the shear force of broiler breast muscle. Notably, the shear force value of the Ber200 group was significantly higher compared to that of the control group (*p* < 0.05).

### Fecal bacterial counts

**Figure 5:**
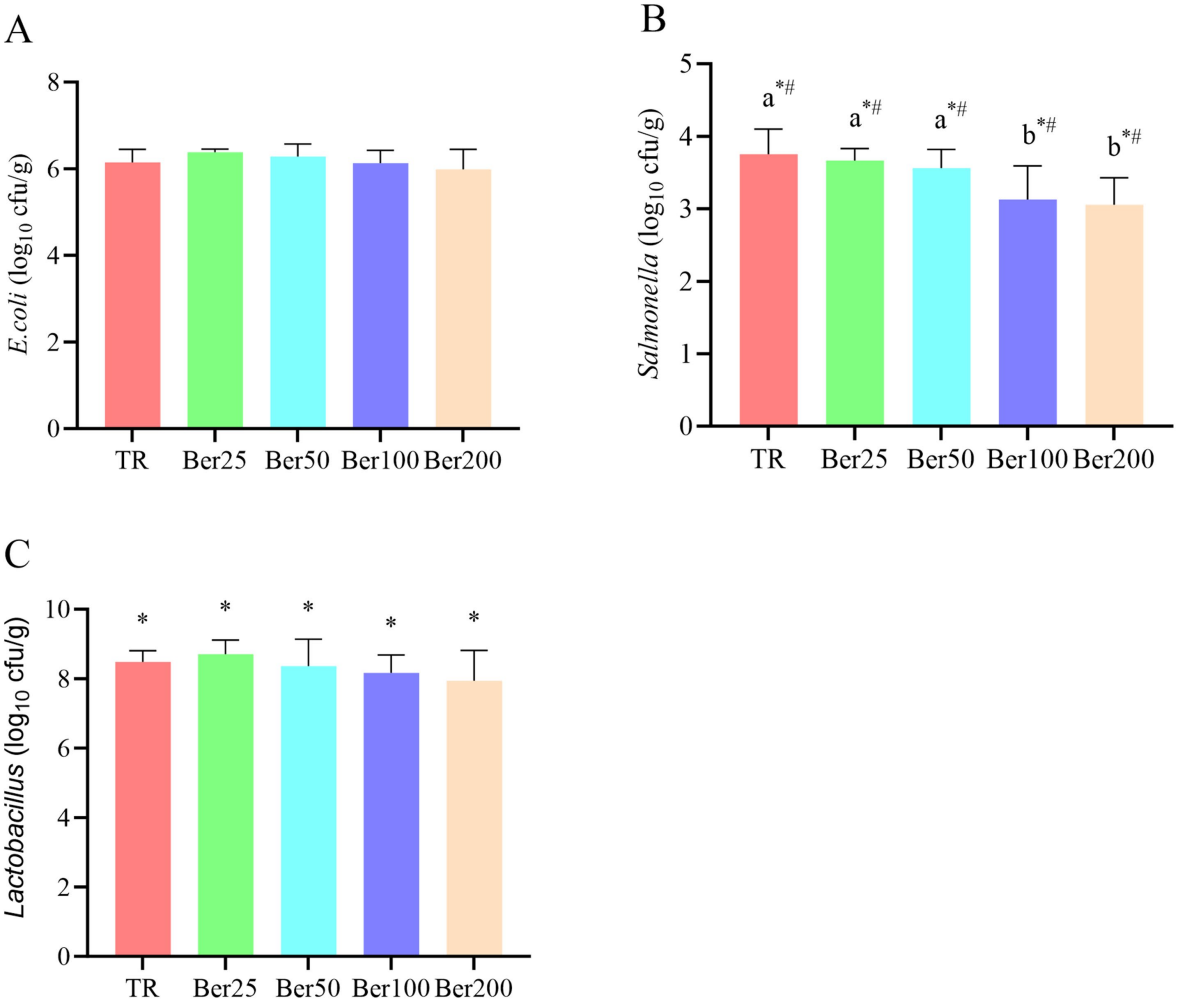
Impact of BBH on the fecal microbiota of AA+ broilers. **(A)** *E. coli*, **(B)** *Salmonella*, and **(C)** *Lactobacillus*. ^a,b^Different superscripts in different groups indicate significant differences (*p* < 0.05). “*” indicates a significant linear effect among these groups (*p* < 0.05). “#” indicates a significant quadratic effect among these groups (*p* < 0.05). No “a, b,” “*,” or “#” indicates that the difference is not significant (*p* > 0.05). The pen was considered the experimental unit for all analyses (*n = 6*).

As depicted in Figure 5, dietary supplementation of varying dosages of BBH led to a significant reduction in the quantity of *Salmonella* in broiler feces. Moreover, both linear and quadratic effects were evident (*p* < 0.05). Regarding the number of *Lactobacillus* in broiler feces, a linear influence was present (*p* < 0.05), but the differences among the groups failed to reach statistical significance (*p* > 0.05).

### Correlation analysis

**Figure 6:**
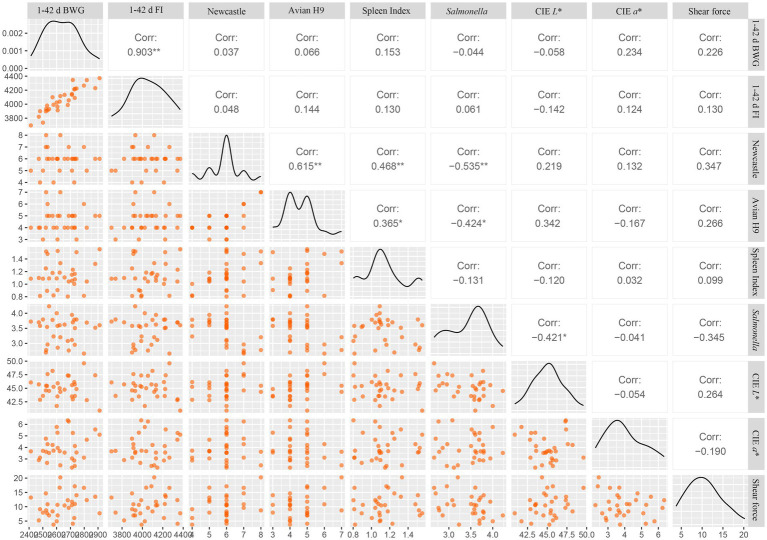
Joint *t* analysis chart of correlations and distributions of growth, immunity, pathogen, and meat quality indicators in AA+ broilers. “*” indicates a significant difference (*p* < 0.05). “**” indicates an extremely significant difference (*p* < 0.001).

Figure 6 depicts the correlation analysis of the test indicators that exhibit significant differences among the various broiler groups at 42 days of age. The findings indicate that the BWG from 1 to 42 days is highly significantly positively correlated with the FI from 1 to 42 days (*r* = 0.903, *p* < 0.001). Specifically, the Newcastle level in the serum demonstrates a highly significant positive correlation with both the avian H9 level (*r* = 0.615) and the spleen index (*r* = 0.468) (*p* < 0.001), while it shows a highly significant negative correlation with the quantity of *Salmonella* in the feces (*r* = −0.535, *p* < 0.001). Additionally, the avian H9 level in the serum is significantly positively correlated with the spleen index (*r* = 0.365, *p* < 0.05) and significantly negatively correlated with the quantity of *Salmonella* in the feces (*r* = −0.424, *p* < 0.05). Moreover, the quantity of *Salmonella* in the feces is significantly negatively correlated with the CIE L* value of the broiler breast muscle (*r* = −0.421, *p* < 0.05).

## Discussion

### Growth performance: stage-specific effects and dose–response relationships

The present study demonstrated that berberine hydrochloride (BBH) exerted no significant effects on growth parameters in broilers during the early growth phase (1–21 days of age) but significantly increased body weight gain (BWG) and feed intake (FI) in the late growth phase (22–42 days of age) and over the entire experimental period. Moreover, the optimal growth-promoting effect was observed at a dose of 50 mg/kg BBH, displaying a typical quadratic response. This stage-specific effect is consistent with the report by Liu et al. (19) on the growth-promoting effects of plant extracts in broilers during the late growth phase and also aligns with the finding that low-dose berberine in piglets requires intestinal maturation to exert its biological effects (20). The lack of significant effects in the early phase may be attributed to the incomplete development of intestinal morphology and function in young broilers, including low villus height, crypt depth, and digestive enzyme activities (21), as well as an unstable intestinal microbiota (11), which limits the promoting effect of BBH on nutrient utilization. As the intestinal tract matures, BBH may exert its growth-promoting effects by improving intestinal barrier function, regulating the expression of genes related to energy metabolism (22), or enhancing nutrient absorption efficiency.

Conversely, the growth-promoting effect was reduced in the high-dose (200 mg/kg) group, suggesting the possible occurrence of hormesis or increased metabolic burden. This finding is consistent with the report by Rad et al. (23) that high-dose berberine may interfere with cellular metabolism and exert toxic effects, and it also agrees with studies showing that other alkaloid-based additives inhibit animal growth at high doses (24). Notably, despite the significant increases in FI and BWG, the feed conversion ratio (FCR) remained unchanged, indicating that BBH improved growth performance without compromising feed utilization efficiency. This provides support for the feasibility of BBH as a growth promoter in broiler production.

### Immune-enhancing effects and their underlying mechanisms

Dietary supplementation with berberine hydrochloride (BBH) linearly increased spleen and bursa of Fabricius indices, indicating a promoting effect on the development of central immune organs. At 42 days of age, Newcastle disease and avian influenza H9 antibody levels rose with increasing BBH dosage, peaking in the 200 mg/kg group. This finding is consistent with the study by Yang et al. (25), reporting that berberine enhances immune responses in broilers via the NF-κB signaling pathway, and it also aligns with the conclusion of Yang et al. (14) that berberine activates B-cell receptor signaling and promotes antibody production in a *Salmonella* infection model.

Although the magnitude of the increase in antibody levels was moderate, it still reflects a positive regulatory effect of BBH on humoral immunity, which may be associated with mechanisms such as regulating T-cell differentiation and modulating cytokine balance (6). Notably, antibody levels in the present study were negatively correlated with fecal Salmonella counts, suggesting that BBH may indirectly inhibit intestinal pathogen colonization by enhancing systemic immune responses. This effect is consistent with the findings of Xiao et al. (7), who demonstrated that BBH enhances host defense function via the p38 MAPK pathway, thereby promoting pathogen clearance. However, it must be emphasized that the antibodies measured in the present study are only indicative markers of immune responses and do not directly equate to protective efficacy against virulent wild-type virus challenge. Further studies are required to validate its immune-protective efficacy in combination with challenge trials.

### Dual effects on meat quality: increased lightness and decreased tenderness

Dietary supplementation with berberine hydrochloride (BBH) significantly increased the lightness (CIE L* value) of broiler breast muscle, with the most pronounced effect observed in the 100 mg/kg group. The improvement in meat color may be attributed to reduced intestinal pathogenic bacteria (7), decreased oxidative stress (26), and enhanced stability of muscle pH, which is consistent with the findings of Gao et al. (27) that improved intestinal health can positively influence meat quality.

However, high-dose BBH (200 mg/kg) resulted in a significant increase in shear force, indicating decreased muscle tenderness. This negative effect may be associated with increased cross-linking of muscle collagen, inhibited protease activity, or redistribution of energy metabolism—previous studies have confirmed that the reprogramming of energy and protein metabolism directly affects meat quality (28). Although limited literature is currently available on the direct effects of berberine on broiler muscle structure, Zhu et al. (29) found that berberine can regulate plasma protein metabolism indices, suggesting that it may indirectly affect muscle protein turnover and structural composition by modulating protein metabolism. Meanwhile, high-dose alkaloid compounds may disturb the balance of protein metabolism and impair myofibrillar degradation (consistent with the metabolic regulatory role of berberine), which also indirectly supports this hypothesis. Nevertheless, this association still requires further validation with direct detection data in muscle tissue. Therefore, although BBH can improve meat color overall, its dose-dependent negative effect on tenderness determines that excessively high doses (e.g., 200 mg/kg) are not advisable for practical application. Moderate-to-low doses (50–100 mg/kg) can better maintain the eating quality of meat while promoting growth and enhancing immunity.

### Selective modulation of the intestinal microbiota

Dietary supplementation with berberine hydrochloride (BBH) linearly and quadratically decreased fecal *Salmonella* counts, while exerting no significant inhibitory effect on *Lactobacillus* counts. Some studies have reported that BBH may slightly increase *Lactobacillus* abundance (14, 29), and this discrepancy may be associated with BBH dosage, experimental duration, or the physiological status of broilers. Overall, BBH exhibited a selective modulatory property characterized by “suppressing pathogens while preserving beneficial bacteria”. Not only is this property consistent with the finding that berberine exerts a specific disruptive effect on the cell membranes of Gram-negative bacteria (30), but it can also be explained by the molecular mechanism whereby BBH activates innate immunity via the p38 MAPK pathway and targets the inhibition of pathogenic bacterial colonization (7). Meanwhile, it aligns with the core mechanism of BBH as an antibiotic alternative—exerting effects by regulating intestinal microbiota balance rather than through broad-spectrum bacteriostasis (19, 31).

However, it should be noted that the present study only performed culture-based counting for three bacterial species, which failed to comprehensively reflect the overall structural and functional changes of the intestinal microbiota. Previous studies using 16S rRNA gene sequencing have confirmed that berberine-related compounds can significantly alter the cecal microbiota composition of broilers (e.g., increasing the abundance of phylum *Bacteroidetes* and genus *Lactobacillus*, and reducing the proportion of harmful bacteria) and regulate microbial metabolic functions (29). Future studies are warranted using 16S rRNA gene sequencing or metagenomic approaches to systematically elucidate the effects of BBH on intestinal microbiota diversity, core microbiota interactions, and functional pathways in broilers, providing more comprehensive microbiota-level evidence for its application as an antibiotic alternative.

## Conclusion

This study examined the effects of dietary berberine hydrochloride (BBH, 25/50/100/200 mg/kg) on 1-day-old AA+ broilers over 42 days, with the 50 mg/kg BBH group (Ber50) showing the best comprehensive performance. In terms of growth, Ber50 did not affect 1–21-day performance but significantly boosted body weight gain and feed intake during 22–42 days and the full period (quadratic effect), outperforming other groups. Immune and microbiota indicators: BBH improved immune organ indices and reduced fecal *Salmonella* levels, while Ber50 avoided the toughness associated with 200 mg/kg BBH and the limited growth promotion of lower doses. In conclusion, a dosage of 50 mg/kg BBH balances growth performance, intestinal health, and meat quality, making it the optimal dose for AA+ broiler production, providing strong support for the use of BBH as a green feed additive.

## Data Availability

The raw data supporting the conclusions of this article will be made available by the authors, without undue reservation.

## References

[1] Mehdi Y Létourneau-Montminy MP Gaucher ML Chorfi Y Suresh G Rouissi T . Use of antibiotics in broiler production: global impacts and alternatives. Anim Nutr. (2018) 4:170–8. doi: 10.1016/j.aninu.2018.03.002, 30140756 PMC6103476

[2] Arsène MMJ Davares AKL Viktorovna PI Andreevna SL Sarra S Khelifi I . The public health issue of antibiotic residues in food and feed: causes, consequences, and potential solutions. Vet World. (2022) 15:662–71. doi: 10.14202/vetworld.2022.662-671, 35497952 PMC9047141

[3] Zhou X Wang J Lu C Liao Q Gudda FO Ling W. Antibiotics in animal manure and manure-based fertilizers: occurrence and ecological risk assessment. Chemosphere. (2020) 255:127006. doi: 10.1016/j.chemosphere.2020.127006, 32417517

[4] Obeidat MD Alkhateeb MEM Jawasreh KI Riley DG Al Sukhni IA. Herbal extract dietary supplementation effect on growth performance and meat quality in broiler raised under two stocking densities. Sci Rep. (2024) 14:18633. doi: 10.1038/s41598-024-68138-8, 39128913 PMC11317506

[5] Sevillano F Blanch M Pastor JJ Ibáñez MA Menoyo D. Effects of olive pomace and spice extracts on performance and antioxidant function in broiler chickens. Animals. (2025) 15:808. doi: 10.3390/ani15060808, 40150337 PMC11939207

[6] Qin Z Tang R Liang J Jia X. Berberine, a natural alkaloid: advances in its pharmacological effects and mechanisms in the treatment of autoimmune diseases. Int Immunopharmacol. (2024) 137:112422. doi: 10.1016/j.intimp.2024.112422, 38880024

[7] Xiao Y Cui Y Zhang Y Fu W Liu Y Liu F. Berberine hydrochloride enhances innate immunity to protect against pathogen infection via p38 MAPK pathway. Front Immunol. (2025) 16:1536143. doi: 10.3389/fimmu.2025.1536143, 40092994 PMC11906452

[8] Meng J Wang W Ding J Gu B Zhou F Wu D . The synergy effect of matrine and berberine hydrochloride on treating colibacillosis caused by an avian highly pathogenic multidrug-resistant *Escherichia coli*. Poult Sci. (2024) 103:104151. doi: 10.1016/j.psj.2024.104151, 39137499 PMC11372597

[9] Huang YQ Liu JL Chen GX Shen DT Zhu W Chen XL . Berberine enhances intestinal mucosal barrier function by promoting vitamin D receptor activity. Chin J Integr Med. (2024) 30:143–51. doi: 10.1007/s11655-023-3547-x, 37046128

[10] Ehteshamfar SM Akhbari M Afshari JT Seyedi M Nikfar B Shapouri-Moghaddam A . Anti-inflammatory and immune-modulatory impacts of berberine on activation of autoreactive T cells in autoimmune inflammation. J Cell Mol Med. (2020) 24:13573–88. doi: 10.1111/jcmm.16049, 33135395 PMC7754052

[11] Wang Y Shou JW Li XY Zhao ZX Fu J He CY . Berberine-induced bioactive metabolites of the gut microbiota improve energy metabolism. Metabolism. (2017) 70:72–84. doi: 10.1016/j.metabol.2017.02.003, 28403947

[12] Pan H Bi J Hu H Huang Y Li A Zhang H . Chinese herbal medicine improves antioxidant capacity of chicken liver at high stocking density involved gut-liver microbiota axis based on multi-omics technologies. Poult Sci. (2025) 104:105015. doi: 10.1016/j.psj.2025.105015, 40106906 PMC11964641

[13] Dehau T Cherlet M Croubels S Van De Vliet M Goossens E Van Immerseel F. Berberine-microbiota interplay: orchestrating gut health through modulation of the gut microbiota and metabolic transformation into bioactive metabolites. Front Pharmacol. (2023) 14:1281090. doi: 10.3389/fphar.2023.1281090, 38130410 PMC10733463

[14] Yang L Sun J Yang T Zhang X Xu C Wei Y . Therapeutic effects and mechanisms of berberine on enteritis caused by *Salmonella* in poultry. Front Microbiol. (2024) 15:1458579. doi: 10.3389/fmicb.2024.1458579, 39664055 PMC11631916

[15] NRC. Nutrient requirements of poultry. Washington, DC, USA: National Academic Press (1994).

[16] Dall'Ara P Lauzi S Filipe J Caseri R Beccaglia M Desario C . Discrepancy between in-clinic and haemagglutination-inhibition tests in detecting maternally-derived antibodies against canine parvovirus in puppies. Front Vet Sci. (2021) 8:630809. doi: 10.3389/fvets.2021.630809, 33732742 PMC7959788

[17] Lee D Lee HJ Jung DY Kim HJ Jang A Jo C. Effect of an animal-friendly raising environment on the quality, storage stability, and metabolomic profiles of chicken thigh meat. Food Res Int. (2022) 155:111046. doi: 10.1016/j.foodres.2022.111046, 35400431

[18] Dang DX Yong MK Kim IH. Effects of a root extract from *Achyranthes japonica* Nakai on the growth performance, blood profile, fecal microbial community, fecal gas emission, and meat quality of finishing pigs. Livest Sci. (2020) 239:1–5. doi: 10.1016/j.livsci.2020.104160

[19] Liu M Zhou J Li Y Ding Y Lian J Dong Q . Effects of dietary polyherbal mixtures on growth performance, antioxidant capacity, immune function and jejunal health of yellow-feathered broilers. Poult Sci. (2023) 102:102714. doi: 10.1016/j.psj.2023.102714, 37172360 PMC10196799

[20] Mouchtoglou C Cherlet M Dehau T Aluwe M Ducatelle R Goossens E . A low dose of berberine is metabolized in weaned piglets without major changes to gut morphology or gut microbiota. Animals (Basel). (2025) 15:2450. doi: 10.3390/ani15162450, 40867778 PMC12383199

[21] Ravindran V Abdollahi MR. Nutrition and digestive physiology of the broiler chick: state of the art and outlook. Animals. (2021) 11:2795. doi: 10.3390/ani11102795, 34679817 PMC8532940

[22] Cheng H Liu J Tan Y Feng W Peng C. Interactions between gut microbiota and berberine, a necessary procedure to understand the mechanisms of berberine. J Pharm Anal. (2022) 12:541–55. doi: 10.1016/j.jpha.2021.10.003, 36105164 PMC9463479

[23] Rad SZK Rameshrad M Hosseinzadeh H. Toxicology effects of *Berberis vulgaris* (barberry) and its active constituent, berberine: a review. Iran J Basic Med Sci. (2017) 20:516–29. doi: 10.22038/IJBMS.2017.8676, 28656087 PMC5478780

[24] Wang J Deng L Chen M Che Y Li L Zhu L . Phytogenic feed additives as natural antibiotic alternatives in animal health and production: a review of the literature of the last decade. Anim Nutr. (2024) 17:244–64. doi: 10.1016/j.aninu.2024.01.012, 38800730 PMC11127233

[25] Yang L Liu G Liang X Wang M Zhu X Luo Y . Effects of berberine on the growth performance, antioxidative capacity and immune response to lipopolysaccharide challenge in broilers. Anim Sci J. (2019) 90:1229–38. doi: 10.1111/asj.13255, 31264347

[26] Klandorf H Dartigue V. Oxidative stress and plasma ceramides in broiler chickens. Front Physiol. (2024) 15:1411332. doi: 10.3389/fphys.2024.1411332, 39077757 PMC11284268

[27] Gao CQ Shi HQ Xie WY Zhao LH Zhang JY Ji C . Dietary supplementation with acidifiers improves the growth performance, meat quality and intestinal health of broiler chickens. Anim Nutr. (2021) 7:762–9. doi: 10.1016/j.aninu.2021.01.005, 34466680 PMC8379291

[28] Nihashi Y Shinji S Umezawa K Shimosato T Ono T Kagami H . Myogenetic oligodeoxynucleotide complexed with berberine promotes differentiation of chicken myoblasts. Anim Sci J. (2021) 92:e13597. doi: 10.1111/asj.13597, 34309956

[29] Zhu C Huang K Bai Y Feng X Gong L Wei C . Dietary supplementation with berberine improves growth performance and modulates the composition and function of cecal microbiota in yellow-feathered broilers. Poult Sci. (2021) 100:1034–48. doi: 10.1016/j.psj.2020.10.071, 33518062 PMC7858044

[30] Yang X Wang Y Li L Tang D Yan Z Li M . Berberine and its nanoformulations and extracts: potential strategies and future perspectives against multi-drug resistant bacterial infections. Front Microbiol. (2025) 16:1643409. doi: 10.3389/fmicb.2025.1643409, 40964668 PMC12436466

[31] Zhou W Asif A Situ C Wang J Hao H. Multiple target and regulatory pathways of berberine. Phytomedicine. (2025) 146:157030. doi: 10.1016/j.phymed.2025.157030, 40763599

